# The effects of health literacy in influenza vaccination competencies among community-dwelling older adults in Hong Kong

**DOI:** 10.1186/s12877-020-1504-5

**Published:** 2020-03-14

**Authors:** Fan Zhang, Peggy Pui-Lai Or, Joanne Wai-Yee Chung

**Affiliations:** Department of Health and Physical Education, The Education University of Hong Kong, Room 10, 2nd Floor, Block D, 10 Lo Ping Road, Tai Po, New Territories Hong Kong

**Keywords:** Health literacy, Vaccination, Older adults, European health literacy survey, Disease prevention, Health promotion, Health care

## Abstract

**Background:**

Poor health literacy was found to be one of the key barriers in older adults’ disease prevention practice. However, it has still been unclear how different processes in health literacy play a role in older adult’s vaccination-related competencies. By adopting the European Health Literacy Survey (HLS-EU), the current study aimed to examine older adults’ competences in accessing, understanding, evaluating and applying health information, as well as how they are related to perceived difficulties in vaccination-related practices. .

**Methods:**

With a cross-sectional design, a quantitative exploratory study was conducted using structured questionnaires. Four-hundred and 86 community-dwelling older adults aged 65 and above were recruited from non-government organizations in Hong Kong. Health literacy was measured by the Chinese version of 47-item HLS-EU (HLS-Asia-Q), which assesses the competences in accessing, understanding, evaluating and applying health information across the domains of health care, disease prevention, and health promotion. Linear regression was performed to test the association between different aspects of health literacy and vaccination-related information processing.

**Results:**

The findings showed that the health literacy of Hong Kong older adults has been limited, particularly in information appraisal. Poorer competences in accessing and appraising health information were associated with greater difficulties in making vaccination decision.

**Conclusions:**

By identifying the health literacy processes associated with vaccination, our findings suggested that health-promotion programs strengthening the appraisal and comparison of vaccination information should be provided for the general public. Meanwhile, health professionals and mass media should reduce the complexity when delivering the health messages, and make it easier for older adults to access and comprehend, thus increasing the inclination to take vaccine and preventing the spread of communicable diseases.

## Background

As an umbrella concept, health literacy encompasses people’s knowledge and competences to access, understand, appraise and apply health information in order to make judgements and take decisions in everyday life concerning healthcare, disease prevention and health promotion to maintain or improve quality of life during the life course [1]. Previous studies have found the social gradient of health literacy is as a pivotal factor contributing to the disparities in health status and preventive care utilization [2, 3]. With higher level of health literacy, many diseases, health conditions, and injuries could be prevented or eliminated by early detection, and preventive measures (e.g., vaccination), especially for the vulnerable populations, i.e., older adults.

However, so far the level of health literacy among older adults has been relatively limited, which could thereby lead to delayed or poor preventive health practice. In a European survey conducted with 8,000 older people aged above 76, it was found that the majority showed insufficient or problematic health literacy [1]. Meanwhile, a national survey conducted in the United States (*N* = 18,000) revealed that the low level of health literacy was associated with a decrease in the utilization of preventive health measures among the older adults aged 65 years or above [4]. Poor health literacy was also found to account for the education- and race– related disparities in health status and receipt of influenza vaccination among the older people [3]. However, most of the existing research has been conducted in western societies, and the evidence in Chinese sample has remained limited. With a large-scale survey in Beijing, Zhang et al., have found that the level of communicable disease health literacy (CDHL) was inadequate, especially among those who were aged above 60, doing manual works and illiterate [5].

As a conclusion, given that health literacy plays a critical role in promoting individual’s preventive health behaviors, the limited health literacy of the older adults may suggest high risk for emerging infectious diseases or health conditions. For example, according to the Advisory Committee on Immunization Practices (ACIP), adults aged 50 or above were recommended to take annual influenza immunization [6]. As a cost-effective approach [7], influenza vaccination could reduce the probability of nosocomial infections, out-patient visits and economic loss [8]. However, the rate of receiving influenza vaccination among those aged above 65 in Hong Kong was only 27% in 2015 [9], and 29% in 2016 [10], which was much lower than the target rate, i.e., 90%, for older adults identified by the Healthy People 2010 [11]. To understand this low uptake rate, previous studies have investigated the roles of socioeconomic disparities, negative attitudes of patient and provider, as well as access barriers. After adjusting for demographics factors, physician visits, and health status, individuals with limited health literacy were consistently found to be less likely to receive influenza immunization [12].

However, it remains unclear how various processes of health literacy function in people’s prevention health process such as influenza vaccination uptake. According to a systematic review [13], existing literature has been mainly focused on the functional health literacy referring to the capability to transmit real information to vaccinations, with little considerations of individual’s cognitive and literacy skills of extracting and applying relevant information. In addition, the instruments used in prior studies often assessed health literacy as a unidimensional concept by stressing reading skills. As a result, little evidence were found on how other capacities and processes were associated with older adults’ vaccination practice. As a comprehensive concept, multiple processes were involved in health literacy, i.e., accessing, understanding, appraising and applying various health information. A new tool assessing health literacy, namely the European Health Literacy Survey (HLS-EU) [14], has been developed to address these processes. In particular, HLS-EU covers three domains including health care, health promotion, and disease prevention. By acknowledging health literacy as a dynamic process with complex interrelations, this instrument extends the existing literature by underscoring the interactive and critical health literacy in term of using information or knowledge as a basis for making health decisions [15]. By adopting the translated version of HLS-EU [16], the current study aimed to fill the research gap by investigating: 1) The health literacy level across multiple domains among community-dwelling older adults in Hong Kong; 2) how different processes of health literacy affect their vaccination practice.

## Methods

### Design

Using a cross-sectional design, a quantitative exploratory study was conducted with face-to-face interviews and structured questionnaires.

### Sample

Four hundred and 86 community-dwelling older adults were recruited randomly from community health centers, and social and health centers for the older people all over Hong Kong. Recruitment criteria included: 1) aged 65 or above; 2) being able to understand Chinese (Mandarin or Cantonese); 3) no history of cognitive impairment or hearing dysfunctions. Considering the relatively low level of literacy of the older cohort in Hong Kong, the questionnaires were administered by a trained research assistant in Cantonese. Ethical approval for the study was obtained from the Human Research Ethics Committee of the Education University of Hong Kong. A written consent was obtained from the participants before taking part in the study.

### Measures

Demographic information included sex (1 = male, 2 = female), age, education (1= “primary education or below”, 2= “secondary education or above”), marital status, employment, living status, and health conditions were collected. For individual’s health condition, the presence of 28 physical symptoms and chronic disease were evaluated, including hypertension, high blood cholesterol, high blood lipid, diabetes, cardiovascular disease, heart failure, respiratory disease, asthma, thyroid disease, liver disease, rheumatism, arthritis, osteoporosis, other musculoskeletal disease, cancer, depression, anxiety disorder, mood disorder, other mental health problem, eating disorder, alcoholism, drug abuse, reproductive disease, hearing impairment, visual impairment, limb loss, and other. The total number of present health condition was obtained to indicate individual’s general health.

The Asian version (HLS-Asian-Q) of the 47-item HLS-EU was adopted to measure health literacy. HLS-EU was based on the conceptual model developed within the HLS-EU consortium [17], and pretested for understandability and completeness using focus groups in Greece, Ireland, and Netherlands. The translated versions have been validated in 6 Asian countries including Taiwan, Indonesia, Kazakhstan, Malaysia, Myanmar, and Vietnam [16]. In Taiwan, the Chinese version reached an excellent Cronbach Alpha (=0.96), suggesting a good reliability. The questionnaire included three domain-specific scales of health literacy, i.e., health care, disease prevention, and health promotion. In addition, it also contains four information-processing specific scales to access the perceived difficulties in a) seeking, finding, and obtaining health information (13 items); b) understanding, or comprehending health information (11 items); c) appraising, interpreting, filtering, or evaluating health information (12 items); and d) applying or communicating and using the information to maintain and improve health (11 items). With a 4-point Likert scale, the responses ranged from “1” (very difficult) to “4” (very easy), and “5” (do not know) was coded as missing value. The total score of general HL was obtained, which was transformed into an index with the formula suggested by the European Health Literacy Project [I = (Mean − 1) * 50/3] [18]. After the transformation, the HL index ranges from 1 to 50, with higher score indicating greater HL. The Cronbach alpha was 0.986 suggesting good internal consistency. With the cutoff value used in previous literature [17], a HL index of 0 to 25 may indicate “inadequate” perceived health literacy, and a value from 26 to 33 was defined as “problematic”; while an HL index in the range of 34 to 42 indicated “sufficient”, and a value above 42 may suggest “excellent” perceived health literacy. The same cutoff value was also applied to different domains of information processing [19].

As for vaccination-related HL, it was measured by the competences of accessing, understanding, assessing, and applying information related to taking vaccinations for preventing disease. In particular, four items from the “disease prevention” dimension were used to ask participants how difficult they perceived about “finding information about vaccinations and health screenings that you should have”, “understanding why you need vaccinations”, “judging which vaccinations you may need”, and “deciding if you should have a flu vaccination”.

### Statistical analysis

To explore the reliability of HLS-EU in Hong Kong sample, Cronbach’s alphas were calculated for each subscales. All the subscales showed satisfactory internal consistency of the items, with a Cronbach’s α greater than 0.9. With correlation analysis, we have identified the covariates that are correlated with the outcome variable, and adjusted them in further analysis. Multiple linear regression was conducted to address the effects of various health literacy in vaccination-related information processing. All the analysis was performed with SPSS 24.0.

## Results

Four hundred and 86 older adults aged from 65 to 93 have completed the questionnaires. 39.9% of the participants were male, and the majority had an educational level of no formal education or primary school (67.8%), were living with family member (81.1%), and were married (64.5%), for details, see Table 1. As for the competences of finding, understanding, appraising, and applying health information, the rating per item was close to 3, in the middle between “fairly difficult” and “fairly easy”. It was noteworthy that the lowest rating was found in the domain of evaluating information on health-care (2.31), suggesting greater perceived difficulty. Meanwhile, the highest rating was on applying health-care information (3.01), indicating the perceived difficulty/competence vary across the specific domains and processes of health literacy.

**Table 1:** The descriptive results of demographic variables and health literacy

			Mean (SD)	
Age (range: 65–93)			74.03 (7.02)	
Gender (% of male)			39.9%	
Education				
Primary education or below			67.8%	
Secondary education or above			32.2%	
Competence domain			Mean (SD)	Mean (SD) per item (range:1–4)
Health care	Finding information	4	10.46 (2.72)	2.62 (0.68)
	Understanding information	4	10.67 (2.64)	2.67 (0.66)
	Evaluating information	4	9.26 (2.97)	2.31 (0.74)
	Applying information	4	12.03 (2.56)	3.01 (0.64)
	Total (range: 16–64)	16	42.11 (9.74)	2.63 (0.61)
Disease prevention	Finding information	4	9.89 (2.89)	2.47 (0.72)
	Understanding information	3	8.25 (2.18)	2.75 (0.73)
	Evaluating information	5	12.16 (3.68)	2.43 (0.75)
	Applying information	3	8.01 (2.06)	2.67 (0.69)
	Total (range: 15–60)	15	38.23 (10.22)	2.55 (0.68)
Health promotion	Finding information	5	12.57 (3.43)	2.52 (0.69)
	Understanding information	4	10.13 (2.55)	2.53 (0.64)
	Evaluating information	3	8.03 (2.28)	2.68 (0.76)
	Applying information	4	10.67 (2.70)	2.67 (0.67)
	Total (range: 16–64)	16	41.87 (10.32)	2.62 (0.65)

Based on the transformed index of HL, the participants were grouped into four categories: “inadequate”, “problematic”, “sufficient”, and “excellent” (see Table 2). In particular, 50.9% of the participants reported inadequate health literacy, and 25.5% showed problematic HL, suggesting that the prevalence of limited health literacy among older adults was quite high. It is note that among those whose education level was low (no formal education or some primary school), approximately 90% showed limited HL. As for different domains of information processing, evaluating information showed the highest rate of “limited health literacy” (84.1%), and applying information showed the lowest (63.9%). The results indicated that older adults perceived the most difficulties in interpreting, filtering, judging and evaluating health-related information, e.g., “judge the advantage and disadvantage of different treatment options”. Meanwhile, they reported the lowest difficulties in applying HL, referring to the ability to communicate and use the information to maintain and improve health, e.g., “follow instructions from your doctor or pharmacist”.

**Table 2:** The distribution of health literacy

	Limited				
Health literacy index score	Inadequate (0–25)	Problematic (26~33)	Total	Sufficient (34~42)	Excellent (> 43)
Finding information	55.7%	27.7%	83.5%	10.9%	5.6%
Understanding Information	50.7%	26.6%	77.4%	16.4%	6.2%
Evaluating information	63.2%	20.9%	84.1%	10.2%	5.7%
Applying information	40.1%	23.8%	63.9%	28.3%	7.8%
General HL	50.9%	25.5%	76.4%	13.2%	10.4%

For immunization, 44.7% of the participants found it “very difficult” or “fairly difficult” to find information about vaccinations and health screenings they need; 36% reported difficulties in understanding why they need vaccinations. Over 55% perceived that it was difficult to judge which vaccinations they may need, and 37.1% found it hard to decide if they should have a flu vaccination. In line with the above findings, the results suggested that older adults perceived the greatest difficulty regarding vaccination practice in evaluating the effects or selecting the type of vaccination that they need. The resulted confusions may cause hesitation or even prevent older adults from taking vaccination.

By adding the scores of the four items related to vaccination, a composite score of vaccination-related HL was generated, which was entered into our linear regression model as the outcome variable. As shown by the correlation analysis (see Table 3), vaccination-related HL was related with age, education, and health condition, therefore, we have included these three as covariates in regression. After excluding the vaccination-related items, total scores were generated for four types of health-information processing competence. The regression model showed that being female (B = 1.11, SE = 0.41, t = 2.70, *p* = 0.008), higher education (B = 1.39, SE = 0.22, t = 6.26, *p* < .001), and fewer health conditions (B = − 0.49, SE = 0.14, t = − 3.42, *p* = .001) were positively associated with greater vaccination-related HL. However, after entering the score of health literacy in finding, understanding, evaluating, and applying information into the regression model, the main effects of education and health condition were not significant. Meanwhile, being female (B = 0.48, SE = 0.22, t = 2.14, *p* = .03), younger age (B = − 0.03, SE = 0.02, t = − 1.99, *p* = .049), greater competence of finding (B = 1.17, SE = 0.49, t = 2.40, *p* = .02) and appraising information (B = 1.82, SE = 0.48, t = 3.83, *p* < 0.001) were associated with higher level of vaccination-related HL, see Table 4.

**Table 3:** The correlation matrix between vaccination-related HL and predictors

	Gender	Age	Edu	Health condition	Finding information	Understanding information	Evaluating information	Applying information
Vaccination-related HL	−0.40	−0.19^**^	0.41^**^	−0.31^**^	0.84^**^	0.85^**^	0.85^**^	0.75^**^
Gender	1							
Age	0.00	1						
Edu	−0.21^**^	− 0.14^**^	1					
Health condition	0.15^**^	0.22^**^	−0.22^**^	1				
Finding Information	−0.01	− 0.30	0.53^**^	− 0.35^**^	1			
Understanding information	−0.10	−0.18^**^	0.47^**^	−0.41^**^	0.91^**^	1		
Evaluating information	−0.13^*^	−0.22^**^	0.45^**^	−0.42^**^	0.91^**^	0.87^**^	1	
Applying information	0.01	−0.10	0.34^**^	−0.18^**^	0.85^**^	0.86^**^	0.75^**^	1

**Table 4:** Regression model predicting vaccination-related health literacy

	B	S.E.	Standardized B	t	*P*-value
Gender	.48	.22	.08	2.14^*^	.04
Age	−.03	.02	−.08	−1.99^*^	.049
Education	.19	.14	.07	1.39	.17
Health condition	−.02	.08	−.01	−.24	.81
Finding information	1.17	.49	.27	2.40^*^	.02
Understanding information	.32	.49	.07	0.66	.52
Evaluating information	1.82	.48	.42	3.89^***^	.000
Applying information	.56	.45	.12	1.25	.21

The outcome variable: Vaccination-related health literacy. “*”: *P* < .05; “**”: *P* < .01; “***”: *P* < .001

## Discussion

Although prior research has investigated the contributing factors for people’s preventive practice against communicable diseases such as influenza, most of which have been focused on the attitude toward immunization, and addressed health literacy as a unidimensional rather than multifaceted concept. By adopting HLS-Asian-Q, an instrument validated across various European and Asian countries, the current study examined the health literacy distribution among older adults in Hong Kong, and how it was associated with the competences in processing vaccination-related information.

Our findings showed that the level of health literacy among older adults has been limited, such that across multiple domains, over 50% of the participants reported a limited level of health literacy, this is similar to the HL level found in other Asian countries. A recent systematic review showed that across the studies conducted in five Southeast Asian countries, the prevalence of limited health literacy ranged from 1.6 to 99.5%, with an average level of 55.3% [20]. The lowest HL was found in evaluating health information, which was consistent with the previous studies in other populations using HLS-EU. For example, in a Dutch sample appraising information was also perceived to be the most difficult [26]. It could be due to that appraising and interpreting doctor’s messages, as well as comparing different treatment or prevention methods, demand greater cognitive ability and resources, thus becoming particularly difficult for older adults. Moreover, the rapidly increased information glut in the media channels such as internet or smart phone apps could be overwhelming and too complicated for older adults to handle [21].

In line with this, the greatest difficulty perceived in vaccination-related items was also found in the information appraisal process. Previous literatures on the associated factors of vaccinations among Hong Kong older people showed that vaccination uptake inclination was predicted by various factors, such as the perceptions of side effects, perceptions of effective durations, and the accessibility of vaccination [22]. Therefore, the comparisons of different vaccinations by reviewing various aspects could be a complex process, especially for those having declined cognitive function. Our analysis has further revealed that when including different competences of information processing in the model, only better competence of finding and evaluating information were associated better vaccination-related capacity. Therefore, promoting individual’s information accessibility and appraisal should be given particular weight in developing screening and intervention programs to promote disease prevention. Also, limited competence in accessing and appraising information should serve as a screening indicator for high-risk older adults.

By identifying that evaluating and interpreting health information are the weakest spot in general HL and vaccination-related process, our findings have significant implications for future interventions promoting older adults’ preventive health practice. As suggested by Rowlands et al., a mismatch between the complexity of health materials and the information processing competence commonly exists in nowadays interventions [23], and this is particularly true for the individuals with lower education. In our sample, over 60% of the participants only have limited education, i.e., no formal education or some primary school, which may amplify the mismatch between the complexity of vaccination-related information and the competence to evaluate this. For future health-literacy promotions, educations programs could be provided to help older adults communicate and appraise health information. Topics such as which aspects should be focused on, or how to compare different vaccinations or treatments should be covered. For health professionals, communication workshops should be organized on how to deliver clear messages regarding the positive aspects of vaccination, which is tailored for older adults to understand. For the society level, as advocated by the World health organization (WHO), an HL-friendly environment should be developed. So far, Hong Kong government has launched the Vaccination Subsidy Scheme (VSS), in which citizens aged above 50 years are entitle to receive subsidized influenza vaccination, and the subsidy was increased to HKD $210 per dose of seasonal influenza vaccination [24]. However, in addition to financial support, our findings suggested that advocacy through the mass-media with reduced complexity, should also be offered to promote public’s uptake of disease prevention measures and treatments [25].

Despite the in-depth insights on HL and its effects from our findings, there were several limitations to be acknowledged. First, lacking direct measures of vaccination uptake prevented us from testing the association between various HL processes and behavioral outcomes. Therefore, although it is plausible that increasing older adults’ competence in information appraisal would reduce the perceived difficulties in processing vaccination-related information, further study are needed to test the direct effects of this in raising vaccination uptake rate. Another limitation is that without longitudinal data, it is difficult to draw clear link between health literacy and preventive health practice. In fact, most existing literature in health literacy has been limited by the cross-sectional data, and cohort study should be conducted to explore how various HL influences older adult’s vaccination in a long term.

## Conclusion

By adopting a multifaceted conceptual model of health literacy, we have assessed Hong Kong older adults’ competences in accessing, understanding, appraising and applying health information, and tested how it was associated with the vaccination-related health competence. According to the findings, the health literacy level among the majority older adults has remained limited, and the competence of appraising health information was the lowest. In addition, limited competence in accessing and appraising health information were associated with greater perceived difficulties in making vaccination decision. The results shed lights on vaccination promotion programs, particularly for older male with poorer health condition, assistance should be provided to help them overcome the obstacles in finding, evaluating and comparing health information, esp. vaccination-related. Meanwhile, health professionals and mass media should reduce the complexity when delivering relevant messages, in order to make it easier for older adults to make health-related decisions. Our findings provide insights for developing programs for enhancing health literacy at individual, institutional and social level, aiming to prevent the spread of infectious disease, and reduce the morbidity and mortality rate in our society.

## Data Availability

The dataset used in the current study is available from the corresponding author on reasonable request.

## Abbreviations

ACIP: Advisory Committee on Immunization Practices
CDHL: Communicable disease health literacy
HL: Health literacy
HLS-EU: European Health Literacy Survey
WHO: World health organization
